# An Item-Level Analysis for Detecting Faking on Personality Tests: Appropriateness of Ideal Point Item Response Theory Models

**DOI:** 10.3389/fpsyg.2019.03090

**Published:** 2020-01-22

**Authors:** Jie Liu, Jinfu Zhang

**Affiliations:** ^1^School of Mathematics and Statistics, Southwest University, Chongqing, China; ^2^Faculty of Psychology, Southwest University, Chongqing, China

**Keywords:** item parameter, ideal point model, faking detection, item response theory, personality tests, appropriate measurement

## Abstract

How to detect faking on personality measures has been investigated using various methods and procedures. As previous findings are mixed and rarely based on ideal point item response theory models, additional research is needed for further exploration. This study modeled the responses of personality tests using ideal point method across instructed faking and honest responding conditions. A sample of undergraduate students participated the within-subjects measures to examine how the item location parameter derived from the generalized graded unfolding model changed, and how individuals’ perception about items changed when faked. The mean test scores of faking group was positively correlated to the magnitude of within-subjects score change. The item-level analysis revealed both conscientiousness items (18.8%) and neuroticism items (50.0%) appeared significant shifts on item parameters, suggesting that response pattern changed from honest to faking conditions. The direction of the change appeared both in positive and negative way, demonstrating that faking could increase or decrease personality factor scores. The results indicated that the changes of perceptions on items could be operated by faking, offering some support for the ideal point model to be an adequate measure for detecting faking. However, the findings of diagnostic accuracy analysis also implied that the appropriateness of ideal point models for detecting faking should be under consideration, also be used with caution. Implications, further research directions, and limitations are discussed.

## Introduction

For many years, faking on personality measures has been perceived as a response distortion or intentional dissimulation. From theoretical perspective, it is well known that the measurement validity of the tests would be significantly affected due to faking, which can negatively impact the quality of the potential personality measures (Topping and O’Gorman, 1997; Stark et al., 2001; Pauls and Crost, 2004, 2005; Holden, 2008; Komar et al., 2008; Buehl et al., 2019). In practical contexts, the typical case is that the candidates who want to improve their chance to be accepted to a job are more likely to fake, even if without any help, they still try to find a way to bring the answers closer to the expectations of the organizations. However, the decision is therefore effected when substantial proportions of applicants would be incorrectly admitted as increasing the likelihood that an organization would hire the fakers (Rosse et al., 1998; Donovan et al., 2014; Niessen et al., 2017). Additionally, even non-real-life-applicants under experimental conditions also can fake when instructed to do so (Thumin and Barclay, 1993; Dalen et al., 2001; Mueller-Hanson et al., 2003; Nguyen et al., 2005; Griffith et al., 2007; Day and Carroll, 2008; Berry and Sackett, 2009; Buehl et al., 2019). Thus, there has been a considerable research interest focused on detecting faking using various methods and procedures.

Many methodologies and techniques have been developed for detecting response distortion over the years, for example, machine learning models, reaction times, regression analysis, etc. (Dunn et al., 1972; Sellbom and Bagby, 2010; Jiménez Gómez et al., 2013; Monaro et al., 2018; Roma et al., 2018; Mazza et al., 2019). Still, there is a concern about the perceptions and interpretations of the change on items due to intentional dissimulation. From an item-level perspective, the changing-item paradigm (Zickar and Robie, 1999) posits that not the standing on the latent trait changes when individuals fake, but the item locations on the continuum that change. In other words, when response distortion occurs, the individuals’ level of the latent trait is fixed without the impact of faking, but the items will be positioned a higher or lower standing on the latent continuum than what is actually possessed. In this case, when the difference of item locations between faking situation and honest situation is captured (i.e., assessed at the item level), the fakability would be identified.

**FIGURE 1:**
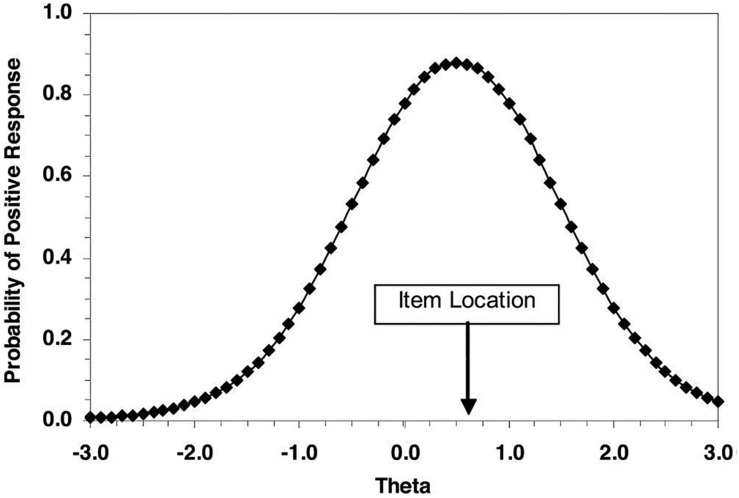
Example of item response function for an ideal point response process.

The research following the changing-item paradigm has often employed differential item functioning (DIF) techniques to address changes over items. As item response theory (IRT) provides a formal statistical model for the relationship between the item response and the latent characteristic, IRT-based DIF is deservedly appropriate for modeling the change of item locations over different responding conditions (Zickar and Robie, 1999; Stark et al., 2001). To describe how people respond to personality measures, the ideal point response process assumes that individuals will have a higher probability to endorse an item that is closer to their “true” latent levels (Roberts, 1996; Roberts and Laughlin, 1996). Specifically, an item response function (IRF) is shown in Figure 1 (Stark et al., 2006). For example, on a measure of conscientiousness (i.e., θ), the agreement probability (i.e., vertical axis) on a statement will be the highest when the item locates nearest the true level of conscientiousness (i.e., horizontal axis). When the distance between conscientiousness level and item location increases, an individual will less likely endorse the item. The generalized graded unfolding model (GGUM) is used as the ideal point model in past years (Roberts and Laughlin, 1996; Roberts et al., 2000). There has already been many previous research that identified advantages of the GGUM in working with personality and attitude data, including the use of understanding faking (Stark et al., 2006; Chernyshenko et al., 2007; Weekers and Meijer, 2008; Tay et al., 2009; Carter and Dalal, 2010; O’Brien and LaHuis, 2011; Speer et al., 2016).

In this study, we performed an item-level analysis to investigate the valence of ideal point IRT models that focus on how perceptions of personality items change when individuals are responding honestly or faking. The within-subjects design was employed to form the comparison groups, under which participants completed both conscientiousness and neuroticism scales. In summary, it can be expected that there is an overall tendency to response distortion that is reflected in different conditions of responding. The hypothesis concerns that different groups of subjects differ in their pattern of selecting options regarding to instructed faking and honestly responding sessions. It is hypothesized that not only the change of test scores can be significantly identified with faking condition, but also the item locations would shift with a dishonest response pattern and the shifts can be examined. Finally, whether the GGUM is adequate for detecting faking needs to be under consideration with caution.

## Methods

### Participants

Respondents consisted of 568 undergraduate students from four Chinese colleges. They volunteered for the study and received extra credit in exchange for their participation. Approximately 78.4% of the participants were female, the average age was 19.84 years (SD = 1.11 years), and non-psychology students. In total, 499 valid cases remained in conscientiousness factor, 547 remained in neuroticism factor. The subjects were excluded from data analysis for two reasons: (a) only one or two response options were selected for all the items (i.e., straight-column answers), and (b) pairwise deleted the data that without an identifying number.

### Design

The response instructions were the within-subjects factor in both experimental sessions. At Time 1, about half of the sample was randomly assigned to respond to the questionnaires honestly, while the other half was assigned to complete the questionnaires with fake instructions. At Time 2, respondents received the opposite set of instructions.

### Procedure

The study was approved by the Institutional Review Board of the Southwest University of China. All participants provided written informed consent after being fully informed of the research procedure.

The questionnaires were administered in paper-and-pencil version in classrooms. The instructions for the honest condition were as follows:

Please complete this personality inventory as honestly as you can. There are no good or bad answers to the items. It is very important that you respond to this survey by describing yourself as you really are and not as you want to be or as you want others to see you.

The instructions for the faking-good condition were as follows:

Imagine that you are applying for a job you really want. Please complete this personality inventory to increase your chances of being hired. To try to give a good impression to the organization, you should present yourselves as the candidates think the organization would like, regardless of your truthful opinions.

After a retest interval of 3 weeks, the second session was the same as the first one except that participants received the other set of response instructions.

### Measures

The International Personality Item Pool (IPIP) is a public-domain measure of the Five-factor model of personality. The IPIP conscientiousness and neuroticism factors are two core personality characteristics that more likely susceptive related to faking (Topping and O’Gorman, 1997; McFarland and Ryan, 2000; Mueller-Hanson et al., 2006; Komar et al., 2008). In this study, the two factors were measured by 20 items from the IPIP, respectively (40 total items). Thus the Conscientiousness Scale and Neuroticism Scale were constructed for measuring the extent to which each item described the respondent on a five-point rating scale ranging from 0 (very inaccurate) to 4 (very accurate). Each scale consists of 10 items that are reverse-coded, and higher composite scores indicate higher levels of traits. The forward–backward procedure was applied to translate the scales from English to Chinese. Participants completed the final Chinese version of the two scales.

### Analyses

Firstly, to examine the veracity of the unidimensional data assumption, a parallel analysis and the matrix of polychoric correlations were performed separately for each response condition on conscientiousness and neuroticism factors. Then, the chi-square test (Drasgow et al., 1995), with the MODFIT program (Stark, 2001) was employed separately for each response condition on both personality factors to examine the fit of the GGUM to the data.

Secondly, the GGUM2004 program (Roberts et al., 2006) was used to obtain the item and person parameters derived from the marginal maximum likelihood estimation method and the expected *a posteriori* estimation method, respectively. Then the GGUMLINK program (Roberts and Huang, 2003) was performed for equating the parameter estimates by transforming the metric of the fake condition group to the same metric of the honest condition group.

Finally, to examine the impact of response distortion on each item, a statistical comparison based on (Scherbaum et al., 2013)’ study was conducted between the GGUM parameter estimates obtained separately under honest and faking conditions. Then we used receiver operating characteristic (ROC) curves analyses to evaluate the diagnostic accuracy of model estimates in detecting faking-induced change^1^.

## Results

### Descriptive Statistics

Descriptive statistics of the row scores of two personality scales in each condition are presented in Table 1. The amount of faking refers as within-subjects change in row scores between two experimental sessions. The intraclass correlation coefficient of the 3-week test–retest was 0.74 (0.70–0.79) for the conscientiousness scale and 0.75 (0.70–0.79) for the neuroticism scale. Under the fake response condition, we observed significant higher scores on conscientiousness (*t*(498) = 5.85, *p* < 0.05, *d* = 0.24), and significant lower scores on neuroticism (*t*(546) = -3.36, *p* < 0.05, *d* = -0.13), compared to the honest response condition, indicating that the faking manipulation was effective. The order effects of response instructions was not statistically significant for conscientiousness (*t*(497) = 0.04, *p* > 0.05, *d* = 0.04), or neuroticism (*t*(545) = 0.72, *p* > 0.05, *d* = 0.06).

**TABLE 1:** Descriptive statistics and reliability of study measures.

	**Honest**			**Faking**			**Amount of faking**			***t***	***d***
	**M**	**SD**	**α**	**M**	**SD**	**α**	**M**	**SD**	**95%CI**		
C	2.33	0.48	0.85	2.45	0.51	0.87	0.12	0.44	0.08–0.16	5.85^∗∗∗^	0.24
N	1.71	0.49	0.83	1.65	0.50	0.83	−0.06	0.44	−0.10−0.03	−3.36^∗∗^	−0.13

*C = conscientiousness; N = neuroticism; amount of faking = change in scores calculated as fake response scores minus honest response scores of pairwise data; α = Cronbach’s α coefficient; 95% CI = 95% confidence interval; *t* = result of *t*-test comparing mean scores in faking and honest response conditions; *d* = Cohen’s *d*, computed using the standard formula of the difference between the means of faking and honest scores divided by the pooled standard deviation. ^∗∗^*p* < 0.01, ^∗∗∗^*p* < 0.001.*

### Correlation Between Faking Scores and Score Changes

According to the results of the correlation matrix (see Supplementary Table 1 in Supplementary Material), scores of personality factors in faking condition were significantly correlated with the magnitude of score change from the faking to honest context, but with moderate correlation coefficients. For conscientiousness, *r* = 0.50 (0.43–0.56, *p* < 0.05), and for neuroticism, *r* = 0.46 (0.41–0.52, *p* < 0.05). This finding suggests that the overall tendency of the change for score elevation is consistent with the test scores related to faking condition, supporting the hypothesis regarding the tendency.

### Test of GGUM Assumptions and Model Fit

One of the assumptions of GGUM is to model data that obtained in unidimensionality personality tests (Roberts et al., 2000). The results of parallel analysis and polychoric correlation coefficients demonstrated that both the conscientiousness and neuroticism data met this assumption. As presented in Table 2, the results of GGUM model fit were reasonably good, except for several items. Hence these four items (“Am always prepared”; “Get chores done right away”; “Do just enough wore to get by”; “Do things according to a plan”) in the Conscientiousness scale under both two conditions were pair-wised excluded from the subsequent analyses for the reliable veracity of model assumptions, as well as a neuroticism item (“Feel comfortable with myself”) under the faking condition, although most IRT estimation procedures are generally tolerant of slight to moderate violations of the unidimensionality assumption (Hulin et al., 1983).

**TABLE 2:** Model fit results of GGUM by scales and conditions.

**Measure**	**Honest**			**Faking**		
	**Number of items with χ^2^/df < 3**	***M***	**SD**	**Number of items with χ^2^/df < 3**	***M***	**SD**
C	17	0.06	0.03	18	0.17	0.15
N	20	0.09	0.13	19	0.07	0.09

*C = conscientiousness; N = neuroticism.*

### Model Parameter Estimates and Shifts in Item Parameter

The item location parameters (i.e., δ) were estimated from GGUM to indicate the location of each item on the latent trait continuum. All of the δ values were positive, as the negatively worded items were recoded and rescored in the positive direction. A test was conducted to identify the differences between the location parameters from the two response groups in order to estimate the shifts. As the differences between item parameters from an IRT model can be considered an effect size (Steinberg and Thissen, 2006), the effect size indicator (i.e., *d*) in this case was the one-to-one difference of the δ (Table 3).

**TABLE 3:** Item parameters for conditions and shifts for each item.

**Measure**	**Item**	**δ**		***t***	***d***
		**Honest**	**Faking**		
C	Waste my time	4.61	6.50	0.19	1.89
C	Pay attention to details	4.66	5.78	0.13	1.12
C	Find it difficult to get down to work	4.06	2.69	–0.22	–1.37
C	Carry out my plans	3.79	4.73	0.30	0.94
C	Do not see things through	4.37	4.98	0.08	0.61
C	Make plans and stick to them	3.71	5.19	0.66	1.48
C	Shirk my duties	3.81	4.36	0.11	0.55
C	Complete tasks successfully	4.30	5.35	0.15	1.05
C	Mess things up	2.52	4.13	5.03^∗∗∗^	1.61
C	Leave things unfinished	3.86	2.78	–0.22	–1.08
C	Am exacting in my work	2.70	4.28	2.87^∗∗^	1.58
C	Don’t put my mind on the task at hand	2.67	5.21	7.26^∗∗∗^	2.54
C	Finish what I start	4.06	4.35	0.05	0.29
C	Make a mess of things	3.46	4.58	0.33	1.12
C	Follow through with my plans	3.96	3.94	0.00	–0.02
C	Need a push to get started	4.37	5.29	0.12	0.92
N	Often feel blue	0.98	0.44	9.00^∗∗∗^	–0.54
N	Seldom feel blue	0.82	0.59	−2.56^∗^	–0.23
N	Dislike myself	0.68	0.37	−4.43^∗∗∗^	–0.31
N	Am often down in the dumps	1.05	2.55	25.00^∗∗∗^	1.50
N	Rarely get irritated	0.21	0.47	2.60^∗∗^	0.26
N	Have frequent mood swings	0.93	1.30	3.70^∗∗∗^	0.37
N	Am not easily bothered by things	0.49	0.63	1.40	0.14
N	Panic easily	0.88	0.86	–0.33	–0.02
N	Am very pleased with myself	0.47	0.34	–1.44	–0.13
N	Am filled with doubts about things	1.31	0.66	−3.82^∗∗∗^	–0.65
N	Am relaxed most of the time	0.65	0.30	−3.50^∗∗∗^	–0.35
N	Feel threatened easily	1.05	0.99	–0.60	–0.06
N	Seldom get mad	0.41	0.49	1.00	0.08
N	Get stressed out easily	1.01	1.14	0.68	0.13
N	Am not easily frustrated	0.56	0.18	−4.22^∗∗∗^	–0.38
N	Fear for the worst	1.19	0.92	–1.08	–0.27
N	Remain calm under pressure	0.47	4.06	29.92^∗∗∗^	3.59
N	Worry about things	1.11	1.07	–0.33	–0.04
N	Rarely lose my composure	0.29	0.08	–1.75	–0.21

*C = conscientiousness; N = neuroticism; δ = item location parameter; *t* = test statistic of the difference between the δ parameters under faking and honest conditions divided by the standard error of the parameter estimates; *d* = the indictor of effect size, calculated as faking δ values minus honest δ values of pairwise data. ^∗^*p* < 0.05, ^∗∗^*p* < 0.01, ^∗∗∗^*p* < 0.001.*

From the table, nearly 20% of conscientiousness items and over 50% of neuroticism items demonstrated statistically significant shifts in the item location parameter. These significant changes occurred in opposite directions in the two personality factors. As the δ is also helpful to index a respondent’s θ level above or below the item location, and the distance between the location of the person and the item, with regard to positive shifts, individuals who were actually at lower levels of this trait tended to select higher order options and appeared as if they were really located on the positive side of the latent trait continuum. Correspondingly, the implication for negative shifts indicated that individuals with high levels of this factor were not likely to select a higher order option and appear as if they were lower on the trait than they really were. These findings supported the hypothesis that the item location could be changed due to response pattern changed and the changes could be modeled using an ideal point IRT model.

### ROC Analyses for Diagnostic Accuracy

Receiver operating characteristic analyses evaluated the shifts of item location parameter for detecting faking-good items versus honest items (see Supplementary Table 2 in Supplementary Material). The area under the curve (AUC) of ROC were 0.74 (SE = 0.12) and 0.64 (SE = 0.13) for conscientiousness factor and neuroticism factor, respectively. Although these AUCs indicated moderate diagnostic accuracy, they are evaluated without statistical significance (*p* > 0.05), suggesting that the effectiveness of the item parameter shifts for examining the faking-induced change of item response pattern was not powerful enough.

## Discussion

The current study used an ideal point IRT model to identify dishonest responses at the item level. We found that the magnitude of score change was positively correlated to the test scores of motived faking group. Parts of the item location parameters derived from the GGUM showed statistically significant shifts across honest and faking conditions in the within-subjects’ response pattern, which indicates that, to some extent, the shifts of item parameters play the role as indicators of faking. Moreover, the accuracy of the indicators was moderately weak for evidencing the appropriateness of ideal point IRT models that used for detect faking.

It was noteworthy that the deltas significantly differed in two response conditions for some items. This demonstrates that operating the response instructions could lead to changes of item positions on the latent trait continuum, and the ideal point IRT model might provide some insight into how faking impacts individuals’ perception of personality items. Specifically, almost all conscientiousness items experienced positive shifts. In this case, individuals with lower levels of the personality characteristic were likely to endorse higher-order options and appear to be higher on the factor than they really were. All the items with significant shifts on the conscientiousness factor showed the same pattern. On the other hand, however, not all the significant neuroticism items followed the same pattern in the direction of the shifts (i.e., negative shifts). The significant reverse shifts demonstrate that the response patterns are complex and sensitive to the characteristic assessed by an item even if such characteristic is not seen as a desirable behavior in the faking condition.

We also found that the magnitude of the shifts was large for many conscientiousness items, whereas it was universally small for neuroticism items. Given that the one-to-one difference of deltas is regarded as an effect size, these values can demonstrate how far apart the item parameters are on the distribution of standardized latent trait. It could be the case that neuroticism is generally not seen as a desirable characteristic and therefore there might not be a uniform perception about these items when respondents fake, so that the direction of distortion varied to generate smaller value of effect size. In addition, the items might show fake in both sides of directions (i.e., positive or negative), which results in counteractions between possible shifts thus less significant shifts in item parameter, and negative impact on accuracy of the IRT-based procedure.

### Implications

Ideal point IRT models (e.g., the GGUM used here) provide an effective means to extend the research on response distortion at the item level. These procedures could quantitatively model the impact of response behavior on personality items and therefore detect the change of response patterns under different response conditions. Positive shifts suggested that the item location on the continuum was higher in the faking condition, whereas negative values indicated that the δ parameter was lower in the faking condition. These findings are consist with the hypothesis that concerning different groups of subjects differ in their pattern of selecting options with respect to different experimental sessions. Not only the change of test scores is significantly identified with instructed faking, but also the item locations shift with a dishonest response pattern and consequently the shifts are examined via an IRT model.

Given that the diagnostic accuracy had appeared unexpected results, the valence of IRT item-analysis might be considered with the issues of appropriateness for ideal point models. It is suggested that if responders compare their self-perception to a certain threshold rather than to the statement’s location, when responding to items, ideal point models should not be used (Brown and Maydeu-Olivares, 2010). Second, focus on the precision of item estimates, it is inherently more difficult to recover true item parameters for ideal point models with the normal probability density function model, if comparing with that for dominance models which derive item estimates with the normal ogive model (Brown and Maydeu-Olivares, 2010). Considering GGUM’s mathematical complexity for estimation difficulties, some studies related to detect faking used other methods, for example, techniques based on reaction times, and scored invalidity scales (Sellbom and Bagby, 2010; Monaro et al., 2018; Roma et al., 2018; Mazza et al., 2019), generally obtained superior accurate outcomes. Finally, practically speaking, the use of ideal point models seems not to result in any improvement for predictive validity, if comparing with dominance models (Zhang et al., 2019). Hence there are still some issues with ideal point models when used for modeling faking response data.

The results of the present study also point to some areas for further research. Firstly, we need to better understand the various direction of the parameter shifts on personality factors. Although the shifts showed a pattern similar to that found in previous research, there is no readily unambiguous explanation for the opposite direction to that being hypothesized. Then, as (Ferrando and Anguiano-Carrasco, 2013) noted, the effectiveness of mixed procedures is higher than that of previous single procedure. The research on faking could benefit from traditional IRT models combined with other recent model-based approaches such as multilevel IRT analysis or mixture IRT models as a starting point.

### Limitations

One potential limitation of this study is the insufficient proportion of double-barreled items and vague quantifiers. If only extreme items are used, dominance and ideal point models will more likely yield a similar fit with nearly monotonical IRFs of personality items (Drasgow et al., 2010). In this case, intermediate statements should be used more frequently for larger effect sizes thereby allowing the researchers to accurately identify an item’s position on the latent continuum underlying faking.

We see an additional limitation regarding the measures of consequent outcomes for the validity of studies under simulated applicant-situations. Generally, these following criterion measures on scales or work performance in real-life context will more accurately predict or estimate the number or percent of the “benefited” items and responders due to faking behavior. It may well be that it provides an available way to examine the internal accuracy and external generalizability.

### Conclusion

Taken together, we find that the test scores in faking condition corresponded with the amount of faking, moreover, the ideal point IRT models in some cases to be an adequate measure for detecting faking at the item level. The shifts of item location parameters offer direct support for the change of individuals’ response pattern due to motivated faking. However, the diagnostic accuracy of the detection is not such ideal so that the usage of ideal point models should be approached with caution. On the whole, this study presents a possible useful method that is worth further investigation.

## Data Availability Statement

The raw data supporting the conclusions of this article will be made available by the authors, without undue reservation, to any qualified researcher.

## Ethics Statement

This studies involving human participants were reviewed and approved by the Institutional Review Board of the Southwest University of China. The patients/participants provided their written informed consent to participate in this study.

## Author Contributions

Both authors contributed to the conception, design of the study, revised the manuscript, read, and approved the submitted version. JZ organized the experimental sessions and led the data collection. JL performed the data analysis and wrote the original draft of the manuscript.

## Conflict of Interest

The authors declare that the research was conducted in the absence of any commercial or financial relationships that could be construed as a potential conflict of interest.

## Footnotes

1 We would like to thank the reviewers for raising this suggestion.

## References

[B1] Berry C. M. Sackett P. R. (2009). Faking in personnel selection: tradeoffs in performance versus fairness resulting from two cut-score strategies. *Person. Psychol.* 62 833–863. 10.1111/j.1744-6570.2009.01159.x

[B2] Brown A. Maydeu-Olivares A. (2010). Issues that should not be overlooked in the dominance versus ideal point controversy. *Indus. Org. Psychol.* 3 489–493. 10.1111/j.1754-9434.2010.01277.x

[B3] Buehl A.-K. Melchers K. G. Macan T. Kühnel J. (2019). Tell me sweet little lies: how does faking in interviews affect interview scores and interview validity? *J. Bus. Psychol.* 34 107–124. 10.1007/s10869-018-9531-3

[B4] Carter N. T. Dalal D. K. (2010). An ideal point account of the JDI Work satisfaction scale. *Pers. Individ. Differ.* 49 743–748. 10.1016/j.paid.2010.06.019

[B5] Chernyshenko O. S. Stark S. Drasgow F. Roberts B. W. (2007). Constructing personality scales under the assumptions of an ideal point response process: toward increasing the flexibility of personality measures. *Psychol. Assess.* 19 88–106. 10.1037/1040-3590.19.1.88 17371125

[B6] Dalen L. H. Stanton N. A. Roberts A. D. (2001). Faking personality questionnaires in personnel selection. *J. Manag. Dev.* 20 729–742. 10.1108/02621710110401428

[B7] Day A. L. Carroll S. A. (2008). Faking emotional intelligence (EI): comparing response distortion on ability and trait-based EI measures. *J. Org. Behav.* 29 761–784. 10.1002/job.485

[B8] Donovan J. J. Dwight S. A. Schneider D. (2014). The impact of applicant faking on selection measures, hiring decisions, and employee performance. *J. Bus. Psychol.* 29 479–493. 10.1007/s10869-013-9318-5

[B9] Drasgow F. Chernyshenko O. S. Stark S. (2010). 75 years after Likert: thurstone was right! *Indus. Org. Psychol.* 3 465–476. 10.1111/j.1754-9434.2010.01273.x

[B10] Drasgow F. Levine M. V. Tsien S. Williams B. Mead A. D. (1995). Fitting polytomous item response theory models to multiple-choice tests. *Appl. Psychol. Measure.* 19 143–165. 10.1177/014662169501900203

[B11] Dunn T. G. Lushene R. E. O’Neil H. F. (1972). Complete automation of the MMPI and a study of its response latencies. *J. Consult. Clin. Psychol.* 39 381–387. 10.1037/h0033855 4405459

[B12] Ferrando P. J. Anguiano-Carrasco C. (2013). A structural model–based optimal person-fit procedure for identifying faking. *Educ. Psychol. Measure.* 73 173–190. 10.1177/0013164412460049

[B13] Griffith R. L. Chmielowski T. Yoshita Y. (2007). Do applicants fake? An examination of the frequency of applicant faking behavior. *Person. Rev.* 36 341–355. 10.1108/00483480710731310

[B14] Holden R. R. (2008). Underestimating the effects of faking on the validity of self-report personality scales. *Pers. Individ. Diff.* 44 311–321. 10.1016/j.paid.2007.08.012

[B15] Hulin C. L. Drasgow F. Parsons C. K. (1983). *Item Response Theory: Application to Psychological Measurement.* Homewood, IL: Dorsey Press.

[B16] Jiménez Gómez F. Sánchez Crespo G. Ampudia Rueda A. (2013). Is there a social desirability scale in the MMPI-2-RF? *Clín. Salud* 24 161–168. 10.5093/cl2013a17 24735833

[B17] Komar S. Brown D. J. Komar J. A. Robie C. (2008). Faking and the validity of conscientiousness: a Monte Carlo investigation. *J. Appl. Psychol.* 93 140–154. 10.1037/0021-9010.93.1.140 18211141

[B18] Mazza C. Monaro M. Orrù G. Burla F. Colasanti M. Ferracuti S. (2019). Introducing machine learning to detect personality faking-good in a male sample: a new model based on Minnesota multiphasic personality inventory-2 restructured form scales and reaction times. *Front. Psychiatry* 10:389. 10.3389/fpsyt.2019.00389 31275176 PMC6593269

[B19] McFarland L. A. Ryan A. M. (2000). Variance in faking across noncognitive measures. *J. Appl. Psychol.* 85 812–821. 10.1037/0021-9010.85.5.812 11055152

[B20] Monaro M. Toncini A. Ferracuti S. Tessari G. Vaccaro M. G. De Fazio P. (2018). The detection of malingering: a new tool to identify made-up depression. *Front. Psychiatry* 9:249. 10.3389/fpsyt.2018.00249 29937740 PMC6002526

[B21] Mueller-Hanson R. Heggestad E. D. Thornton G. C. (2003). Faking and selection: considering the use of personality from select-in and select-out perspectives. *J. Appl. Psychol.* 88 348–355. 10.1037/0021-9010.88.2.348 12731719

[B22] Mueller-Hanson R. A. Heggestad E. D. Thornton G. C. (2006). Individual differences in impression management: an exploration of the psychological processes underlying faking. *Psychol. Sci.* 48 288–312.

[B23] Nguyen N. T. Biderman M. D. McDaniel M. A. (2005). Effects of response instructions on faking a situational judgment test. *Int. J. Select. Assess.* 13 250–260. 10.1111/j.1468-2389.2005.00322.x

[B24] Niessen A. S. M. Meijer R. R. Tendeiro J. N. (2017). Measuring non-cognitive predictors in high-stakes contexts: the effect of self-presentation on self-report instruments used in admission to higher education. *Pers. Individ. Diff.* 106 183–189. 10.1016/j.paid.2016.11.014

[B25] O’Brien E. LaHuis D. M. (2011). Do applicants and incumbents respond to personality items similarly? A comparison of dominance and ideal point response models. *Int. J. Select. Assess.* 19 109–118. 10.1111/j.1468-2389.2011.00539.x

[B26] Pauls C. A. Crost N. W. (2004). Effects of faking on self-deception and impression management scales. *Pers. Individ. Diff.* 37 1137–1151. 10.1016/j.paid.2003.11.018

[B27] Pauls C. A. Crost N. W. (2005). Effects of different instructional sets on the construct validity of the NEO-PI-R. *Pers. Individ. Diff.* 39 297–308. 10.1016/j.paid.2005.01.003

[B28] Roberts J. S. (1996). *Item Response Theory Approaches to Attitude Measurement.* Dissertation, University of South Carolina, Columbia, SC.

[B29] Roberts J. S. Donoghue J. R. Laughlin J. E. (2000). A general item response theory model for unfolding unidimensional polytomous responses. *Appl. Psychol. Measure.* 24 3–32. 10.1177/01466216000241001 31019357

[B30] Roberts J. S. Fang H. R. Cui W. W. Wang Y. J. (2006). GGUM2004: a windows-based program to estimate parameters in the generalized graded unfolding model. *Appl. Psychol. Measure.* 30 64–65. 10.1177/0146621605280141

[B31] Roberts J. S. Huang C. W. (2003). GGUMLINK: a computer program to link parameter estimates of the generalized graded unfolding model from item response theory. *Behav. Res. Methods Instrument. Comput.* 35 525–536. 10.3758/bf03195532 14748497

[B32] Roberts J. S. Laughlin J. E. (1996). A unidimensional item response model for unfolding responses from a graded disagree-agree response scale. *Appl. Psychol. Measure.* 20 231–255. 10.1177/014662169602000305

[B33] Roma P. Verrocchio M. C. Mazza C. Marchetti D. Burla F. Cinti M. E. (2018). Could time detect a faking-good attitude? a study with the MMPI-2-RF. *Front. Psychol.* 9:1064. 10.3389/fpsyg.2018.01064 30090076 PMC6069678

[B34] Rosse J. G. Stecher M. D. Levin R. A. (1998). The impact of response distortion on preemployment personality testing and hiring decisions. *J. Appl. Psychol.* 83 634–644. 10.1037/0021-9010.83.4.634

[B35] Scherbaum C. A. Sabet J. Kern M. J. Agnello P. (2013). Examining faking on personality inventories using unfolding item response theory models. *J. Pers. Assess.* 95 207–216. 10.1080/00223891.2012.725439 23030769

[B36] Sellbom M. Bagby R. M. (2010). Detection of overreported psychopathology with the MMPI-2 RF form validity scale. *Psychol. Assess.* 22 757–767. 10.1037/a0020825 21133544

[B37] Speer A. B. Robie C. Christiansen N. D. (2016). Effects of item type and estimation method on the accuracy of estimated personality trait scores: polytomous item response theory models versus summated scoring. *Pers. Individ. Diff.* 102 41–45. 10.1016/j.paid.2016.06.058

[B38] Stark S. (2001). *MODFIT**: A Computer Program for Model-Data Fit. [Software].* Available at: http://cehs.unl.edu/edpsych/software-urls-and-other-interesting-sites/ (accessed June 14, 2017).

[B39] Stark S. Chernyshenko O. S. Chan K. Lee W. Drasgow F. (2001). Effects of the testing situation on item responding: cause for concern. *J. Appl. Psychol.* 86 943–953. 10.1037/0021-9010.86.5.943 11596810

[B40] Stark S. Chernyshenko O. S. Drasgow F. Williams B. A. (2006). Examining assumptions about item responding in personality assessment: should ideal point methods be considered for scale development and scoring? *J. Appl. Psychol.* 91 25–39. 10.1037/0021-9010.91.1.25 16435936

[B41] Steinberg L. Thissen D. (2006). Using effect sizes for research reporting: examples using item response theory to analyze differential item functioning. *Psychol. Methods* 11 402–415. 10.1037/1082-989X.11.4.402 17154754

[B42] Tay L. Drasgow F. Rounds J. Williams B. A. (2009). Fitting measurement models to vocational interest data: are dominance models ideal? *J. Appl. Psychol.* 94 1287–1304. 10.1037/a0015899 19702371

[B43] Thumin F. J. Barclay A. G. (1993). Faking behavior and gender differences on a new personality research instrument. *Consult. Psychol. J.* 45 11–22. 10.1037/1061-4087.45.4.11 29112881

[B44] Topping G. D. O’Gorman J. G. (1997). Effects of faking set on validity of the NEO-FFI. *Pers. Individ. Diff.* 23 117–124. 10.1016/S0191-8869(97)00006-8

[B45] Weekers A. M. Meijer R. R. (2008). Scaling response processes on personality items using unfolding and dominance models. *Eur. J. Psychol. Assess.* 24 65–77. 10.1027/1015-5759.24.1.65

[B46] Zhang B. Cao M. Tay L. Luo J. Drasgow F. (2019). Examining the item response process to personality measures in high-stakes situations: issues of measurement validity and predictive validity. *Person. Psychol.* 1–28. 10.1111/peps.12353 (in press).

[B47] Zickar M. J. Robie C. (1999). Modeling faking good on personality items: an item-level analysis. *J. Appl. Psychol.* 84 551–563. 10.1037/0021-9010.84.4.551

