# Supplementary material for: An Item-Level Analysis for Detecting Faking on Personality Tests: Appropriateness of Ideal Point Item Response Theory Models
Source: Front Psychol. 2020 Jan 22;10:3090. doi: 10.3389/fpsyg.2019.03090 (PMC6987465; doi:10.3389/fpsyg.2019.03090)
Supplement: Supplementary file 1 [file Table_1.docx]

**S3. Tables and Figures.** The results of correlation analysis and the ROC analysis, and examples of some items for faked response.

Table 1. Correlation Matrix of Test Scores and the Amount of Faking by Scales and Conditions

|  | Honest C(N) | Faking C(N) | Amount of faking C(N) |
| --- | --- | --- | --- |
| Honest C(N) | 1 |  |  |
| Faking C(N) | .59**(.60**) | 1 |  |
| Amount of faking C(N) | -.40**(-.44**) | .50**(.46**) | 1 |

C = Conscientiousness; N = Neuroticism; Amount of faking = change in scores calculated as fake response scores minus honest response scores of pairwise data.

Table 2. Diagnostic Accuracy for the Shifts of Item Location Parameter

| Measure | Area | SE | Asymptotic 95%CI | Asymptotic Sig. |
| --- | --- | --- | --- | --- |
| C | .74 | .12 | .51–.98 | .20 |
| N | .64 | .13 | .39–.90 | .29 |

C = Conscientiousness; N = Neuroticism; 95%CI =95% Confidence Interval.


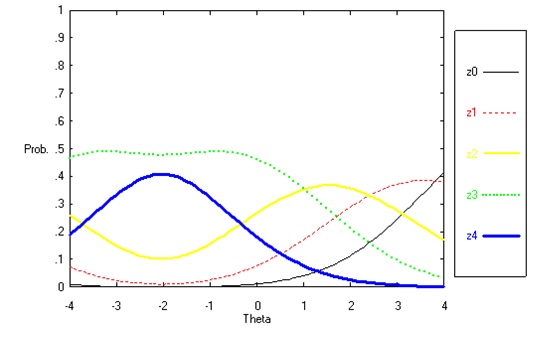

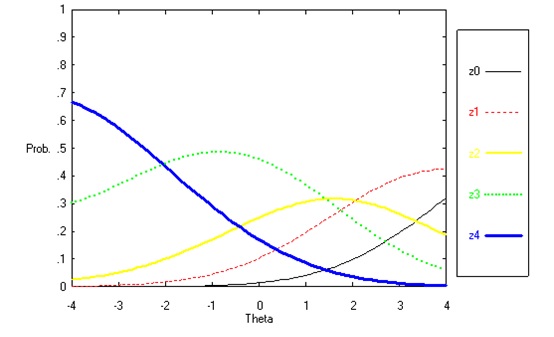
Conscientiousness item “find it difficult to get down to work.”

1. Honest condition (B) Faking-good condition


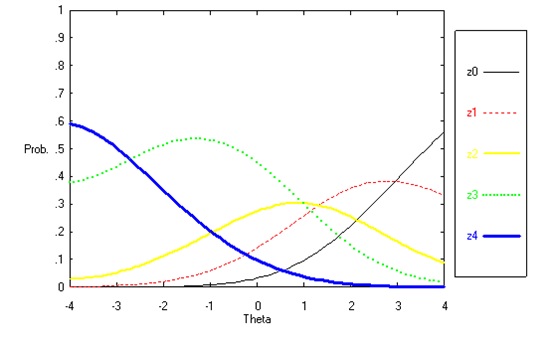
Neuroticism item “fear for the worst.”


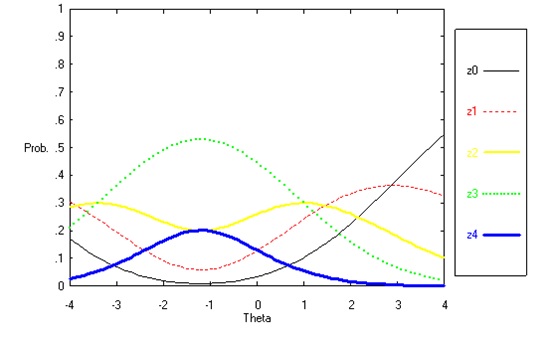
(C) Honest condition (D) Faking-good condition

Figure 1. Option characteristic curve for two sample items that showed item location parameter shift across conditions
